# P302: Securing the venous sampling: a priority for the safety and caregivers of patients in the ped

**DOI:** 10.1186/2047-2994-2-S1-P302

**Published:** 2013-06-20

**Authors:** E Bouvet, E Rouveix

**Affiliations:** 1 GERES, Paris, France

## Objectives

The provision of antiretroviral treatment in developing countries led to the medical care of an increasing number of patients infected with HIV and other viruses and to an increase in invasive procedures practiced by healthcare personnel. Healthcare workers are particularly exposed to the risk of transmission of pathogens during a blood exposure accident (AES). In 2012, the problem of infectious risks related to venepuncture remains a concern for health care in Africa: lack of awareness and lack of resources, inadequate equipment and high prevalence of infection in the population, increase in invasive procedures performed related to extension the management of these diseases and access to health care for the population.

## Methods

GERES, association committed to the prevention of accidental exposure to blood and risk of infection among health care workers, led by France preventive approaches that have proved effective in industrialized countries to reduce this risk. Two symposia on infectious risks in urban care for caregivers in Africa have already been organized in collaboration with the ISSA and ESTHER.

## Results

These meetings have shown a strong mobilization of many caregivers in francophone African countries and pilot studies have shown that venous sampling is the single greatest risk and most common. In addition, feedback from experiences on different sites has already demonstrated the feasibility of securing venous sampling.

The inventory presented at the Lomé symposium in 2012 shows that the venipuncture is still not secure in most health care settings in Africa. The protection of health care with respect to risk of infection, however, is the responsibility of leaders, those who develop national policies, those who manage health care facilities. There is an urgent need to reach an agreement for obtaining the secure hardware cost in developing countries.

## Conclusion

SEVENAF movement (movement to secure the venipuncture in Africa) involving caregivers SSA was created for this purpose.

## Disclosure of interest

None declared

